# Three new emerging subgroups of torque teno sus viruses (TTSuVs) and co-infection of TTSuVs with porcine circovirus type 2 in China

**DOI:** 10.1186/1743-422X-10-189

**Published:** 2013-06-10

**Authors:** Jianbo Liu, Longjun Guo, Long Zhang, Yanwu Wei, Liping Huang, Hongli Wu, Changming Liu

**Affiliations:** 1 Division of Swine Infectious Diseases, State Key Laboratory of Veterinary Biotechnology, Harbin Veterinary Research Institute, The Chinese Academy of Agricultural Sciences, 427 Maduan Street, Nangang District, Harbin 150001, China

**Keywords:** Torque teno sus viruses (TTSuVs), Porcine circovirus type 2, Subgroup, Phylogenetic analysis

## Abstract

**Background:**

Torque teno sus viruses (TTSuVs) are non-enveloped viruses and have single-stranded, negative sense circular DNA genomes and are widely distributed in pigs. But till now, the prevalence of TTSuVs with porcine circovirus type 2 (PCV2) in pig herds of China is not very clear; and the genetic variation among different TTSuVs isolate is very large and need to divide the subgroups. In this study, the co-infection with TTSuVs and porcine circovrius (PCV) in the pig population of China was investigated and the subgroups of all TTSuVs genomes in Genbank were divided.

**Results:**

Results showed that the rate of co-infection with TTSuV1 and TTSuV2 reached 75% in PCV2-positive samples. Also Two TTSuV1 and four TTSuV2 isolates genome sequences were obtained, and the similarity of all TTSuV1 and TTSuV2 genomic sequences in GenBank were compared. Phylogenetic trees indicated that both the TTSuV1 and TTSuV2 sequences could be divided into four genotypes. Interestingly, the sub-genotypes TTSuV1d, TTSuV2c and TTSuV2d exist only in the pig population of China.

**Conclusions:**

This study demonstrates that co-infection with TTSuVs and PCVs is very common in the pig population of China, in which the viruses maybe contribute to clinical diseases cooperatively. In addition, three new subgroups of TTSuVs emerged in China for the first time and a high level of variation among different isolates of TTSuV1 and TTSuV2 was indicated by their genetic diversity.

## Background

Torque teno virus (TTV) was first discovered in a Japanese human patient with post-transfusion hepatitis in 1997 [1]. TTV is a non-enveloped virus that has a single-stranded, negative sense circular DNA genome and belongs to the *Anelloviridae* family, *Iotatorquevirus* genus [2-5]. The virus can infect humans, non-human primates and tupaias and domestic species, including cattle, sheep, pigs, cats, dogs and chickens [6-9]. In humans, TTV infection is ubiquitous and several genogroups have been identified [10,11]. Porcine TTVs (TTSuVs), like their human counterparts, are distributed widely [12], and they can be divided into two distinct species, TTSuV1 [4] and TTSuV2 [13]. Faecal–oral transmission, in addition to vertical transmission, is the most common route of dissemination of TTSuVs [14,15], and TTSuV1 and TTSuV2 have been found in all tissues tested, with variations depending on age, and following similar infection dynamics in all tissues [16]. TTSuVs have been detected in samples from pig herds in many countries, such as Italy, Hungary, France, Spain, Asian countries and North America [8,12,17-23]. It has been demonstrated that the prevalence of both TTSuV1 and TTSuV2 in these countries is very high. In addition, TTSuV1 and TTSuV2 are known to infect both domestic pigs [4,13] and wild boars [24,25]. These reports suggest that TTSuVs are distributed throughout the world.

It is well known that porcine circovirus (PCV) has two genotypes: PCV1 and PCV2. PCV2 can cause postweaning multisystemic wasting syndrome (PMWS), porcine respiratory disease complex (PRDC), granulomatous enteritis, porcine dermatitis and nephropathy syndrome (PDNS), congenital tremors, etc. Former researchers have detected TTSuVs in PCV2 positive samples. And the TTSuVs appear to be ubiquitous in both healthy and sick domestic and wild pigs worldwide and different swine breeds and genders [22], and till now, the role of TTSuVs in the pathogenesis of specific porcine diseases remains debatable. A Korean group reported that they found no significant differences in the viral loads of both TTSuV species between PCV2-negative pigs and pigs affected with PCV-associated diseases (PCVAD) [26]. But in a gnotobiotic pig model, TTSuV1-containing homogenates have been shown to contribute partially to the experimental induction of PMWS and PDNS [27,28]. Although PCV2 is considered to be the primary causative agent for the induction of clinical PMWS or systemic PCVAD [29], it has been observed that PMWS-affected pigs with low or no detectable PCV2 infection had a higher prevalence of TTSuV2 than pigs not affected with PMWS [30,31]. Another study found that TTSuV1 can potentiate PCV2 infections in gnotobiotic swine [27,28], and natural infection with TTSuV1 can suppress the immune response to vaccination against porcine reproductive and respiratory syndrome virus (PRRSV) [32]. Also contradictory results were obtained by a Chinese group [33] that the TTSuVs prevalence or prevalent genotypes of the sick pigs have no difference with healthy pigs, which showed that TTSuVs have no association with PRRSV and swine fever virus. Whether TTSuVs can cause diseases in pig or not requires further study.

The length of the TTSuV1 and TTSuV2 genomes is approximately 2.8 kb and they are organized in at least six open reading frames (ORF1, ORF1/1, ORF2, ORF2/2, ORF1/1/2 and ORF2/2/3) [34] and an untranslated region (UTR) containing regulatory elements believed to be involved in virus replication [35]. The UTR sequence is conserved while the sequence variation of the coding region is very high. Among the several ORF, ORF1 is considered to encode the viral capsid while ORF2 is considered to encode a protein which is used for virus replication [36,37]. Using the full genome sequences of TTSuVs isolates obtained, some researchers have studied the genetic variability of TTSuVs and constructed a phylogenetic tree that divided them into several different clades [35,38].

In Chinese pig herds, the prevalence of TTSuVs and that of co-infection of TTSuVs with PCVs is unknown. In this study, 280 samples from 14 provinces from different areas of China were collected to determine the prevalence of TTSuV1 and TTSuV2 and their co-infection with PCV. In addition, the genome sequences of TTSuVs isolates were analyzed to investigate the diversity and phylogeny of the virus in China.

## Results

### Co-infection of TTSuVs and PCV within pig herds in China

A total of 280 swine inguinal lymph node samples collected from pigs in 14 provinces were tested by PCR for the presence of TTSuVs and PCVs DNA. Overall, of the 280 porcine tissue samples, 170 tested positive for at least one type of TTSuV (Table 1): more specifically, 145 samples were PCR positive for TTSuV1, while 79 were PCR positive for TTSuV2; 51 of these samples contained both TTSuV1 and TTSuV2 genomes. Among the 280 samples, 176 samples were PCV-positive, and 115 of these samples were also PCR-positive for TTSuV1; 105 samples were PCR-positive for PCV2. Fifty-five among all 280 samples were positive for both PCV1 and PCV2. Among the 105 samples positive for PCV2, 80 samples were TTSuV-positive; more specifically, 73 samples indicated co-infection with TTSuV1 while only 30 showed co-infection with TTSuV2.

**Table 1:** Investigation of clinical samples for TTSuV1, TTSuV2, PCV1 and PCV2 by PCR

**District**	**Detection ratio of TTSuV1 and TTSuV2**			**Detection ratio of PCV1 and PCV2**			**Coinfection of TTSuV and PCV2**	
	**TTSuV1**	**TTSuV2**	**TTSuV1 + TTSuV2**	**PCV2**	**PCV1**	**PCV1 + PCV2**	**PCV2 + TTSuV1**	**PCV2 + TTSuV1**
Shanghai	8/12	4/12	4/12	6/12	5/12	3/12	6/12	3/12
Jilin	13/30	3/30	1/30	18/30	11/30	7/30	9/30	3/30
Hebei	3/13	1/13	0/13	2/13	8/13	2/13	0/9	0/9
Beijing	12/19	12/19	10/19	8/19	5/19	4/19	7/19	6/19
Liaoning	2/9	3/9	1/9	1/9	2/9	1/9	1/9	0/9
Shandong	7/12	2/12	2/12	5/12	6/12	4/12	5/12	2/12
Hunan	16/31	10/31	7/31	4/31	10/31	2/31	5/31	4/31
Heilongjiang	76/133	43/133	25/133	51/133	62/133	29/133	33/133	11/133
Others	8/21	1/21	1/21	10/21	6/21	3/21	7/21	1/21
Total	145/280	79/280	51/280	105/280	115/280	55/280	73/280	30/280
	(51.8%)	(28.2%)	(18.2%)	(37.5%)	(41.1%)	(19.6%)	(26.1%)	(10.7%)

Others: the 21 samples were from Sichuan, Hubei, Neimenggu, Jiangsu, Anhui and Zhejiang provinces.

### Cloning of the genomic DNA from TTSuV-positive samples

According to the prevalence results of TTSuVs and PCVs as well as the significant classical PMWS symptom, two isolates of TTSuV1 and four of TTSuV2 were cloned successfully into the vectors.

### DNA sequencing of the cloned viral genomes

Two TTSuV1 (PTTV1-HLJ239, PTTV1-SY13, GenBank no. are JX173481 and JX173482) and four TTSuV2 (PTTV2-BDH278, PTTV2-HUN172-2, PTTV2-HUN172-3 and PTTV2-SH129, GenBank no. are JX173483, JX173484, JX173485 and JX173486) recombinant plasmids were sequenced. All the genomes differed in length. These sequences were submitted to the GenBank database.

### Construction of the phylogenetic tree

**Figure 1:**
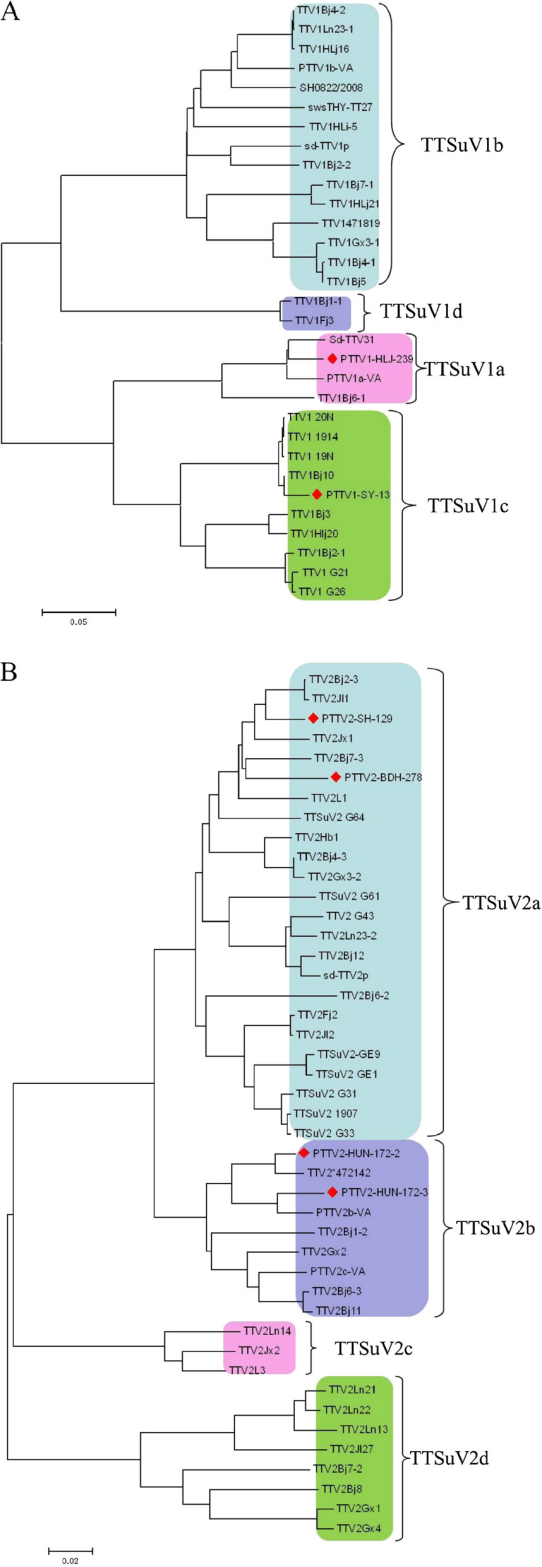
**Phylogenetic tree constructed by the neighbor-joining method based upon the full-length genomic nucleotide sequences of the TTSuV strains.** (**A**) Phylogenetic trees based on 31 full-length sequences of TTSuV1, including 2 strains isolated in this study and 29 strains submitted to GenBank. (**B**) Phylogenetic trees based on 44 full-length sequences of TTSuV2, including 4 strains isolated from this study and 40 strains submitted to GenBank. The tree was constructed using a neighbor-joining algorithm with the MEGA4.0 software. The red color indicates the strains that were isolated in this study.

Sequence analysis was carried out on the genomes of the TTSuV1 and TTSuV2 isolates in GenBank using MEGA 4 software, and two phylogenetic trees were constructed (Figure 1). Analysis of the phylogenetic trees indicated that the TTSuV1 sequences could be divided into four genotypes: TTSuV1a, TTSuV1b, TTSuV1c and TTSuV1d. Among these, TTSuV1b and TTSuV1c were the major groups, while TTSuV1a and TTSuV1d were relatively rare. Similarly, the TTSuV2 sequences could be divided into four genotypes (TTSuV2a, TTSuV2b, TTSuV2c and TTSuV2d), and TTSuV2a was the predominant genotype. Among the all complete sequences of TTSuV1, 20 genomes were derived from China, accounting for 64.5%, and these sequences were distributed among all of the genotypes; five strains were isolated from Spain, which were focused in the TTSuV1c genotype; two sequences were isolated in the USA, located in TTSuV1a and TTSuV1b, respectively. The other four strains were isolated from Canada, Brazil, Japan and Germany. The sequences from Canada, Brazil and Germany belonged to TTSuV1b genotypes while the sequence from Japan was located in TTSuV1a. Thirty-two sequences of TTSuV2 were isolated from China; this variant accounts for 72.7% of all the sequences and was also distributed among all the genotypes. TTSuV2a and TTSuV2b were the predominant genotypes. All the strains isolated from Spain and one strain isolated from Brazil belonged to TTSuV2a, while the three remaining sequences belonged to the TTSuV2b genotype. The other two genotypes, TTSuV2c and TTSuV2d, as well as TTSuV1d appear to exist only in China.

### Homology of TTSuV1 and TTSuV2 sequences

Two TTSuV1 sequences obtained in this experiment were compared with the other sequences for TTSuV1 in GenBank. The similarity ranged from 67.3% to 95.1%. Four TTSuV2 strains with similar genomic nucleic acid sequences showed homology of from 84.7% to 90.4% when compared with 40 TTSuV2 genomic sequences in GenBank used DNAMAN software (Table 2). Within the TTSuV1a group, the similarity was between 87.50% and 95.17%, while in the TTSuV1b group it was 83.81%–99.90% and in the TTSuV1c group 80.26%–97.60%; but only two sequences in TTSuV1a and TTSuV1b groups have been isolated in China. The similarity of the sequences of TTSuV2a ranges between 86.71% and 99.71%, which is similar to TTSuV2b and TTSuV2d, while only two strains obtained in China lie within TTSuV2c. These results demonstrate that the strains within each subgroup also show high variability. Sequences between sub-genotypes of TTSuV1 as well as TTSuV2 were compared by DNAMAN software, and results were shown in Table 3, the similarity between different sub-genotypes of TTSuV1 was among 65.49%–78.19% while the similarity of TTSuV2 was among 69.29%–88.71%.

**Table 2:** The length and similarity of TTSuVs sequences for isolates in different sub-genotypes

**Subgroup**	**Similarity**	**Genomic length**	**Subgroup**	**Similarity**	**Genomic length**
TTSuV1a	87.50%–95.17%	2878–2882 nt	TTSuV2a	86.71%–99.71%	2736–2813 nt
TTSuV1b	83.81%–99.90%	2823–2878 nt	TTSuV2b	88.26%–99.29%	2750–2806 nt
TTSuV1c	80.26%–97.60%	2910–2914 nt	TTSuV2c	93.41%–95.50%	2817–2822 nt
TTSuV1d	98.58%	2897 nt	TTSuV2d	84.86%–98.45%	2822–2834 nt

**Table 3:** The similarity of TTSuVs sequences between sub-genotypes of TTSuV1 and TTSuV2

**Similarity**	**TTSuV1b**	**TTSuV1c**	**TTSuV1d**	**Similarity**	**TTSuV2b**	**TTSuV2c**	**TTSuV2d**
TTSuV1a	67.04%–68.90%	73.85%–78.19%	69.65%–78.12%	TTSuV2a	81.62%–88.71%	73.02%–78.87%	69.26%–78.44%
TTSuV1b		65.49%–70.83%	71.94%–74.59%	TTSuV2b		75.41%–78.88%	71.92%–76.64%
TTSuV1c			68.96%–72.53%	TTSuV2c			77.38%–79.43%

### Deletion and insertion analysis of the TTSuV1 and TTSuV2 genome sequences

**Figure 2:**
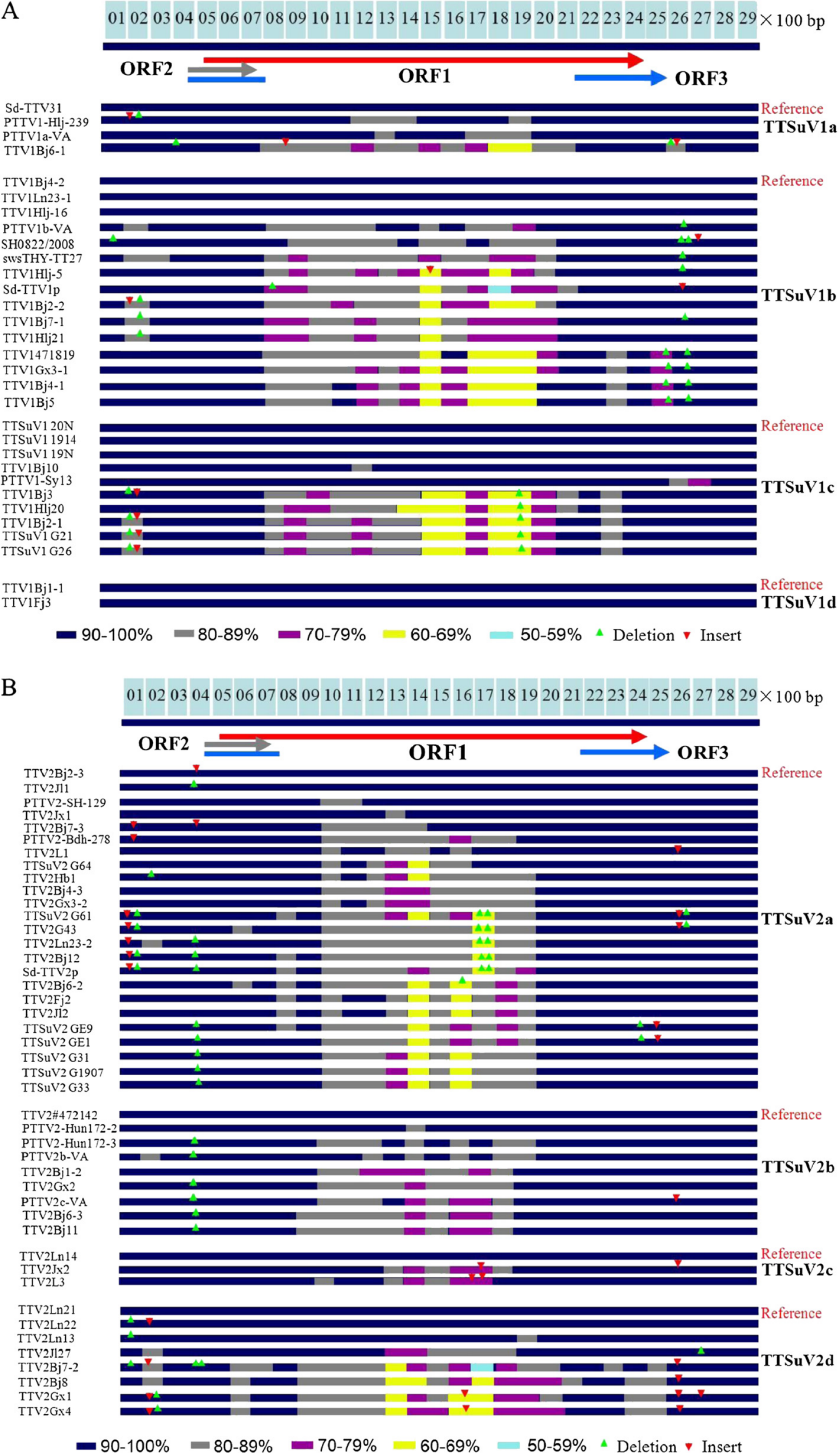
**Schematic diagram of deletion and insertion sites in different sub-genotypes of the TTSuV1 and TTSuV2 genome sequences, with values for similarity.** The genomic similarity of TTSuV1 isolates is indicated by different colors: rectangle blue indicates the similarity is 90–100% while rectangle gray indicates 80–89%; rectangle violet indicates 70–79%; rectangle yellow indicates 60–69% and rectagle aqua indicates 50–59%; arrow red indicates insertion; arrow green indicates deletion. (**A**) In each subgroup, the first sequence of each subgroup, Sd-TTV31, TTV1Bj4-2, TTSuV1 20 N and TTV1Bj1-1, was the reference sequence of TTSuV1a, TTSuV1b, TTSuV1c and TTSuV1d, respectively. Other sequences were compared to the reference. (**B**) In each subgroup, the first sequence of each subgroup, TTV2Bj2-3, TTV2#472142, TTV2Ln14 and TTV2Ln21, was the reference sequence of TTSuV2a, TTSuV2b, TTSuV2c and TTSuV2d, respectively. Other sequences were compared to the reference.

The sequences in each genotype were analyzed using DNAMAN software. As can be seen in Figure 2, the first sequence of each subgenotype was used as the reference and the deletion and insertions occurred at the sequences of the UTR which were the same as those of the other genotypes (TTSuV1b and TTSuV1c). No deletion and insertions appeared in the TTSuV1d genotype, possibly because there were only two sequences. Different colors have been used to show the similarity of different sequences to the reference sequences for each genotype. It can be seen that the variation is very high in the coding region of ORF1. Similar to the situation with TTSuV2, most of the deletions and insertions appeared in the UTR, and low similarities emerged at their coding regions. Among the sequences of the TTSuV1a subgroup, the genome length is between 2878 nt and 2882 nt; the difference in length is caused by the deletion or insertion of a sequence only 5 bp in length. In contrast, in the TTSuV1b subgroup the length of the genome varies from 2823 nt to 2878 nt: the difference in length is greater than for TTSuV1a. In the TTSuV2 subgroups, the difference in length was greater in the TTSuV2a and TTSuV2b subgroups than in TTSuV1a (Table 2), and most of the differences in length occurred at the GC region. Except for the GC region, the differences in genome length among different strains may represent differences of just a few base pairs caused by the deletion of inserts (Figure 2).

## Discussion

The presence of TTV has been reported increasingly around the world, particularly in humans and pigs [30,39]. The present study first investigated co-infection with TTSuVs and PCVs in the pig herds of China. The results showed that the rate of co-infection of TTSuVs and PCV2 was greater than 75%; among the PCV2-positive samples, the prevalence of TTSuV1 was 69% while that of TTSuV2 was 28%. This suggests that TTSuV1 may play an important role, with PCV2, in the induction of PMWS. The result is in accordance with that of Kekarainen et al. [30] and Zhu et al. [33], but the prevalence of TTSuV2 was higher than that of TTSuV1 in the report by Segalés et al. [8] and Huang et al. [40]. Besides, in Liu X et al. [21] study, the prevalence of TTSuV2 equals to that of TTSuV1. This may reflect differences in the environment or the number of samples collected. The 280 samples in this study were collected from 14 provinces of China. Almost every district tested in this study showed the presence of TTSuVs and PCVs, except Zhejiang and Hubei provinces, we could not exclude possibility that TTSuVs and PCVs coexist in these districts due to limited number of samples. The prevalence of TTSuVs around the world is related to fecal–oral transmission, vertical transmission and the use of vaccines, because TTSuVs have been detected in sera, plasma, feces and veterinary vaccines [20,41,42].

The high prevalence of TTSuVs in Chinese pig herds in many regions of China and the presence of a large pig industry in China suggests that much variation may exist among Chinese strains of TTSuVs. Genetic variation among TTSuVs isolates has been reported by researchers worldwide in recent years [25,43]. However, the number of TTSuVs genomes that have been studied is very small; some researchers used partial sequences of TTSuVs genomes [38], and some analyzed only either TTSuV1 or TTSuV2 [25,35]. In former studies, some TTSuVs gene sequences have been amplified using several pairs of primers, followed by assembly of the sequences [4,13,25,43]. Although Cortey et al. [35] used one pair of primers to amplify the genomes of TTSuV1 and TTSuV2, respectively, they did not publish the primers in their report. In this study, we designed one pair of primers for amplification of the TTSuV1 and TTSuV2 genomes, respectively. The primer pairs for the TTSuV1 and TTSuV2 genomes were both located at the conserved UTR region, which made it easier to sequence the genomes of the TTSuVs. Using these two primer pairs, two genomes of TTSuV1 and four genomes of TTSuV2 were isolated, and other sequences obtained from the GenBank were used to analyze genetic variation.

According to the criteria in pairwise sequence comparison (PASC) method and the findings of Huang et al., a TTV type is defined as a group of TTV with 67%–85% nucleotide sequence identity, whereas a TTV subtype may be defined as a group of TTV sequences sharing 85%–95% nucleotide sequence identity. TTV strains sharing more than 95% nucleotide sequence identity may be classified further into variants [35,43,44]. In this study, the most frequent TTSuVs genomes from China were compared and divided into several subtypes. In the phylogenetic tree for TTSuV1, the viruses were divided into four subtypes, TTSuV1a, TTSuV1b, TTSuV1c and TTSuV1d, while TTSuV2 were classified into TTSuV2a, TTSuV2b, TTSuV2c and TTSuV2d. According to the subtypes divided by phylogenetic tree, similarity of sequences in the same subtypes as well as in different subtypes were compared and showed in Table 2 and Table 3. The results are mostly in accordance with the TTSuVs criteria that could be followed to identify the subtypes of TTSuV1 and TTSuV2.

In recent studies, TTSuVs also have been divided into several subtypes. TTSuV1 was divided into four subtypes as described by Cortey et al. [38], they used some sequences that were not TTSuv1 full genomes, and did not show the sequences names or GenBank numbers. TTSuV1 was divided into 3 subtypes (subtype 1a, 1b, 1c). In this research, TTSuV1 was divided into 4 subtypes (TTSuV1a, 1b, 1c and 1d). The TTSuV1a, 1b and 1c subtypes in this study are in accordance with Li et al. [23] study. Furthermore, we also find a new subtype TTSuV1d according to the PASC method that a TTV subtype may be defined as a group of TTV sequences sharing 85%–95% nucleotide sequence identity [43]. TTSuV2 was divided into 7 subtypes (subtype 2a, 2b, 2c, 2d, 2e, 2f and 2g). However, after being compared carefully, all these TTSuV2 subtypes divided by Li et al. [23] were classified into two subtypes in our study: TTSuV2a (subtype 2a, 2d, 2e, 2f and 2g) and TTSuV2b (subtype 2b and 2c). Besides that, the genomes in TTSuV2c and TTSuV2d didn’t emerge in their research. And in this study, the similarity of sequences in TTSuV2a and TTSuV2b subtypes were >86.71% (Table 2), so the subtype 2a, 2d, 2e, 2f and 2g can be classified as TTSuV2a subtype and subtype 2b, 2c can be classified as TTSuV2b subtype according to the PASC method [43].

Earlier research has divided the viruses into only two groups, TTSuV1 and TTSuV2, although some studies have defined several subtypes for each genotype, but based on small sample sizes. Huang et al. [43] divided TTSuV1 into two subtypes, type1a and type1b, while TTSuV2 were classified into three separate subtypes (2a, 2b, 2c). Two subtypes of TTSuVs described by Huang et al. [43], subtype 2b (TTSuV2b-VA) and subtype 2c (TTSuV2c-VA), were located in the same subtypes, the TTSuV2b group. The phylogenetic reconstructions indicate that one TTSuV1 subtypes (TTSuV1d) and two TTSuV2 subtypes (TTSuV2c and TTSuV2d) have been described for the first time in the current study and the sequences in the three subtypes were all come from China which shows that three new subtypes of TTSuVs emerged in China for the first time. The high variation of TTSuVs sequences suggests that the other subtypes may exist in pig herds, and this warrants further study. Although the genomes of different subtypes are not equivalent, the differences are caused mainly by deletion or insertion, especially in the TTSuV2a and TTSuV2b subtypes. The difference in length may be caused by sequencing problems in the GC-rich region of the TTSuV2 genome, although other possible reasons include insertion or deletion.

Also, in this study, although the PTTV2-HUN-172-2 and PTTV2-HUN-172-3 strains were obtained from the same samples, their similarity was only 93.09%. This showed that different strains of TTSuVs can affect the same host, which is in accordance with the study of Huang et al., who isolated four strains of TTSuVs from a single pig [43]. Comparison of the similarity of TTSuV1 and TTSuV2 isolated in China demonstrated the degree of similarity among the strains in different subtypes, as shown in Table 2. In each subtypes, the similarity is within 80%–100%; given that the full genomes are no longer than 3000 nt, this shows that, even in one group the variation is very large. In TTSuVs genomes, the variation is distributed unevenly; the untranslated region is more conserved and the translated region is more variable. Figure 2 show that the ORF1 of both TTSuV1 and TTSuV2 are highly variable in the region from 700 nt to 2000 nt, which suggests that they may give rise to antigenic diversity. A recent report by Huang et al. [43] demonstrated antigenic cross-reactivity between two TTSuV1 isolates: PTTV1a-VA (belongs to TTSuV1a subtype) and PTTV1b-VA (belongs to TTSuV1b subtype) [34]. However, whether other strains in the two subtypes, or TTSuV1a, 1b with TTSuV1c, 1d also have this antigenic cross-reactivity need further study.

However, for limited numbers of TTSuV1 and TTSuV2 genomes available, the exacted definition of subtypes may be postponed. Also, more sequences of TTSuVs genomes should be obtained from different geographic regions and then analyzed for better conclusions on TTSuVs divergence.

## Conclusions

In summary, in the present study co-infection with PCVs and TTSuVs was detected in the pig population of China, and the prevalence of co-infection of TTSuVs and PCV2 is very high. Also, two TTSuV1 and four TTSuV2 genomes have been characterized from the co-infection samples with PCVs and TTSuVs and compared with the genomes available in the GenBank. The genetic variation was analyzed: the results showed that both TTSuV1 and TTSuV2 were divided into four subtypes. And one subtypes of TTSuV1, TTSuV1d, had emerged for the first time together with two new subtypes, TTSuV2c and TTSuV2d, of TTSuV2. The analysis of variation in TTSuVs showed that the variability is focused in the ORF1 region. This study provides valuable information for further study of genetic variation in TTSuVs.

## Materials and methods

### Sample collection

The tissue samples used in the study were obtained from 280 post weaning piglets that had become sick post weaning. The samples were all collected from inguinal lymph nodes which were collected in 14 provinces of China during 2004 to 2011 and stored in a freezer at −80°C until further processing. The geographical origin and the number of samples are summarized (Table 1). The tissue samples used in this study was approved by Harbin Veterinary Research Institute, Chinese Academy of Agricultural Sciences and performed in accordance with animal ethics guidelines and approved protocols. The animal Ethics Committee approval number is Heilongjiang-SYXK-2011-145. To minimize the risk of contamination, each step of the PCR procedure, DNA extraction, DNA amplification and electrophoresis, was carried out in a separate rooms. The DNA from 200 μl of each tissue homogenate was extracted using the TaKaRa MinBEST Viral DNA Extraction Kit Ver.4.0 (Takara Co. Dalian, China) according to the manufacturer’s instructions.

### PCR detection of TTSuV1, TTSuV2, PCV1 and PCV2

In order to evaluate the presence of TTSuV1 and TTSuV2, the collected samples were used in a PCR method that amplifies a non-coding region of the viral genome. The primers were designed using Oligo 6.0 (Table 4). For the PCR reaction, the primers PTTV1-F1 and PTTV1-R1 were used for the detection of TTSuV1, and PTTV2-F1 and PTTV2-R1 were used for the detection of TTSuV2. The reaction conditions were 94°C 5 min; 94°C 30 s, 55°C 30 s, 72°C 30 s for 35 cycles, and a final extension for 10 min at 72°C. The primer pairs PCV1-F1 and PCV1-R1, PCV2-F1 and PCV2-R1 were used for the detection of PCV1 and PCV2, respectively. The reaction conditions were 94°C 5 min; 94°C 30 s, 55°C 30 s, 72°C 30 s (PCV1) or 45 s (PCV2), for 35 cycles, and a final extension for 10 min at 72°C. Each PCR product was run on a 1.8% TAE–agarose gel.

**Table 4:** Primer sequences for detection of PCVs and TTSuVs

**Primer**	**Primer sequence**	**Location (nt)**	**Product size (bp)**
PTTV1-F1	5’-CGGGTTCAGGAGGCTCAAT-3’	8–26	305
PTTV1-R1	5’-GCCATTCGGAACTGCACTTACT-3’	291–312	
PTTV2-F1	5’-TCATGACAGGGTTCACCGGA-3’	1–20	252
PTTV2-R1	5’-CGTCTGCGCACTTACTTATATACTCTA-3’	226–252	
PTTV1-F2	CAGCGGTAGACAGAACTGTCTAGCGACT	328–355	< 3000
PTTV1-R2	TCTGTCTACCGCTGGCGGCATAAACTCA	314–341	
PTTV2-F2	TCGAGCTCCTGAGAGCGGAGTCAAGGGGCCTA	322–353	< 3000
PTTV2-R2	TCGAGCTCCGGCACCCGCCCAGGCGGTTAGAC	300–331	
PCV1-F1	5’-TTGCTGAGCCTAGCGACACC-3’	596–615	350
PCV1-R1	5’-TCCACCTGCTTTCAAATCGGCC-3’	924–945	
PCV2-F1	5’-CAGCAAGAAGAATGGAAGAAGCGGA-3’	56–80	1057
PCV2-R1	5’-CCAGGACTACAATATCCGTGTAACT-3’	1088–1112	

### PCR amplification of genomic DNA

Primers were used to amplify the TTSuV1 and TTSuV2 genomes at their conserved region. The TTSuV1 primers were PTTV1-F2 and PTTV1-R2 while the TTSuV2 primers were PTTV2-F2 and PTTV2-R2 (Table 4). The PCR parameters for both viruses comprised 30 cycles of denaturation at 94°C for 2 min, annealing at 62°C for 30 s and extension at 68°C for 3 min, followed by a final incubation at 68°C for 7 min.

### Cloning of the viral genome

The PCR products from two TTSuV1 and four TTSuV2 isolates from each geographical location were purified using an EZNA™ Cycle-pure kit, following the manufacturer’s instructions. The PCR products were cloned into the pMD18-T vector after an A was added to the products at the ends of PCR fragments. Subsequently, the recombinant plasmids were transformed into *E. coli* TOP10 competent cells. The resulting colonies were screened according to the manufacturer’s instructions. Positive colonies were detected using the PCR protocol described above, except that the first denaturation step was performed at 94°C for 2 min. Plasmid DNA was extracted using the Axygen Plasmid Miniprep Kit (Axygen Co., Hangzhou, China) according to the manufacturer’s instructions; TTSuV1 recombinant plasmids were identified by restriction enzyme analysis with *Apa*lI, *Kpn*I and *Sal*I and TTSuV2 recombinant plasmids were identified by restriction enzyme analysis with *Apa*lI and *Sac*I.

### Sequencing of the full genome of TTSuVs

Plasmids from six different colonies per strain were selected for sequencing at a commercial facility (Sangon Co., Shanghai, China). Both strands of the insert were sequenced at least twice, using the M13 universal primers and other primers that had been designed according to the TTSuVs sequences to obtain the full genome sequences. The sequences of the DNA fragments were assembled using DNAMAN software (version 5.22, Lynnon Biosoft, 1994). The six full genomes of the TTSuVs isolates were submitted to the GenBank database.

### Sequences and phylogenetic analysis

Twenty-nine representative full-length genomes of TTSuV1 and 40 representative full-length genomes of TTSuV2 were downloaded from GenBank database, partial genome of TTSuV1 or TTSuV2 was excluded. The sequences obtained in this study were analyzed using several software programs, including MEGA 4.0, DNAStar, and DNAMAN, to align the sequences, estimate nucleotide distances and diversities (nucleotide distances both between and within genotypes) and assess the phylogenetic relationships by the neighbor-joining method using 1000 bootstrap replicates.

## Competing interests

None of the authors of this paper has a financial or personal relationship with other people or organizations that could inappropriately influence or bias the content of the paper.

## Authors’ contributions

JL organized the whole process, took part in all the experiments and wrote the manuscript. CL designed the whole project. LG, LZ, YW, LH and HW participated in the clinical materials collection. All authors read and approved the final manuscript.
